# Identification of Key Pathways and Genes in the Orai2 Mediated Classical and Mesenchymal Subtype of Glioblastoma by Bioinformatic Analyses

**DOI:** 10.1155/2019/7049294

**Published:** 2019-10-20

**Authors:** Feng Yuan, Li Yi, Long Hai, Yingshuai Wang, Yihan Yang, Tao Li, Luqing Tong, Haiwen Ma, Peidong Liu, Haolang Ming, Bingcheng Ren, Shengping Yu, Yu Lin, Xuejun Yang

**Affiliations:** ^1^Department of Neurosurgery, Tianjin Medical University General Hospital, Tianjin 300052, China; ^2^Laboratory of Neuro-Oncology, Tianjin Neurological Institute, Tianjin 300052, China; ^3^The First Affiliated Hospital of USTC, Division of Life Sciences and Medicine, University of Science and Technology of China, Hefei, Anhui 230001, China; ^4^Department of Internal Medicine III, University Hospital Munich, Ludwig-Maximilians-University, Munich, Germany

## Abstract

**Background:**

Ca^2+^ release-activated Ca^2+^ channels (CRAC) are the main Ca^2+^ entry pathway regulating intracellular Ca^2+^ concentration in a variety of cancer types. Orai2 is the main pore-forming subunit of CRAC channels in central neurons. To explore the role of Orai2 in glioblastoma (GBM), we investigated the key pathways and genes in Orai2-mediated GBM by bioinformatic analyses.

**Methods:**

Via The Cancer Genome Atlas (TCGA), French, Sun, and Gene Expression Omnibus (GEO) (GDS3885) datasets, we collected 1231 cases with RNA-seq data and analyzed the functional annotation of Orai2 by gene ontology (GO) and Kyoto Encyclopedia of Genes and Genomes (KEGG) pathway analysis. Univariate and multivariate survival analyses were applied to 823 patients with survival data.

**Results:**

We discovered that Orai2 was markedly upregulated in GBM compared to normal brain samples and lower-grade gliomas (LGG). Survival analysis found that higher expression of Orai2 was independently associated with a worse prognosis of patients with the classical and mesenchymal subtypes of GBM. Simultaneously, Orai2 expression was higher in tumors of the classical and mesenchymal subtypes than other subtypes and was significantly correlated with classical- and mesenchymal-related genes. GO and KEGG pathway analysis revealed that genes significantly correlated with Orai2 were involved in the JNK pathway. Through screening transcriptomic data, we found a strong association between Orai2 and apoptosis, stemness, and an epithelial-mesenchymal transition- (EMT-) like phenotype.

**Conclusion:**

As a prognostic factor, Orai2 is obviously activated in the classical and mesenchymal subtypes of GBM and promotes glioma cell self-renewal, apoptosis, and EMT-like by the JNK pathway. These findings indicate that Orai2 could be a candidate prognostic and therapeutic target, especially for the classical and mesenchymal subtypes of GBM.

## 1. Introduction

Gliomas are the most common primary intracranial tumors. Glioblastoma multiforme (GBM) is the most aggressive form (WHO grade IV) with high morbidity among different types of gliomas [1]. The standard treatment methods start with maximal surgical resection, followed by radiotherapy with concomitant and adjuvant temozolomide. However, most patients will die within 1 to 2 years. A median progression-free survival of 6.2 to 7.5 months and a median overall survival of 14.6 to 16.7 months have been reported in a clinical trial [2]. One of the reasons for this high mortality is the therapeutic resistance of GBM, mainly due to its diffuse growth, genetic heterogeneity, and microvascular proliferation [3].

The discovery of glioma stem cells (GSCs) is an important event in glioma research, which has profoundly affected our study of GBM patterns, tumor heterogeneity, tumor cell and microenvironment relationships, and treatment strategies [4]. Glioma stem cells, also known as glioma-initiating cells (GICs), are tumor cells with self-renewal and infinite differentiation potential and have a high degree of invasion, migration and tumorigenicity. GSCs maintain the vitality of tumor cell populations through self-renewal and immortalization, and the ability to move and migrate makes it possible to spread tumor cells. GSCs can also be dormant for a long time and have multiple drug-resistant molecules that are insensitive to the external physical and chemical factors that kill tumor cells and are the drivers of tumor progression and recurrence [5].

Epithelial-mesenchymal transition (EMT) is a process in which epithelial properties are impaired and interstitial properties are enhanced [6]. The EMT in GBM has gradually attracted increased attention in recent years. However, what occurs during the progression of GBM is not complete EMT but a transformation process similar to epithelial-mesenchymal transition, called EMT-like, which refers to a process in which the epithelial phenotype is less pronounced and more biased toward the mesenchymal phenotype, mainly manifested by downregulation of epithelial markers such as E-cadherin and increased expression of interstitial markers such as N-cadherin and fibronectin, which leads to a decrease in cell-to-cell adhesion and loss of cell polarity, ultimately enhancing tumor invasion and migration [7]. Therefore, as one of the most challenging malignancies worldwide, effective therapies for GBM are needed.

Since Bailey and Cushing first proposed the systematic classification of neuroepithelial tissue tumors in 1926, the concept of histogenesis has dominated CNS tumor classification for almost a century. The histology-based WHO classification and grading system serves as a “gold standard,” playing an important role in the diagnosis and treatment of CNS tumors. In addition, the pathological diagnostic criteria of gliomas, especially GBMs, which have caused artificial heterogeneity and complexities in investigations, did not have useful effects on clinical management. In 2016, the WHO CNS tumor histology classification (revised version 4) integrated the histological phenotype and gene phenotype for the first time. IDH, 1p/19q, TP53, pTERT, ATRX, and other indicators were included in the integrated diagnostic criteria for gliomas. Since then, the diagnosis and treatment of gliomas have entered the era of molecular typing [8]. However, glioma diagnosis with the integration of histological and gene phenotype information still needs further improvement. Verhaak et al. analyzed the expression profiles of 202 GBMs in The Cancer Genome Atlas (TCGA) and classified GBMs into four subtypes: proneural, neural, classical, and mesenchymal. Each subtype differs greatly in terms of its cellular features, genetic contexts, and signaling pathways involved. In TCGA classification of GBMs, different molecular subtypes do not share the same response and survival benefit to traditional treatment [9]. The molecular typing of GBM makes the inclusion and grouping of target patients in clinical trials more homogenous and balanced. This system also helps to identify important molecular targets in each subtype to design feasible therapeutic strategies.

The c-Jun NH2-terminus kinases (JNKs) are a family of stress-activated serine threonine protein kinases of the MAPK pathway. Mitogen-activated protein kinases (MAPKs) are serine-threonine protein kinases that regulate various cellular activities including proliferation, differentiation, apoptosis, survival, inflammation, and innate immunity. Compromised MAPK signaling pathways have also been found to play key roles in the pathogenesis of various diseases including cancer and neurodegenerative disorders. Three distinct genes, known as JNK1, JNK2, and JNK3, encode ten splice variants. Four JNK1 isoforms, four JNK2 isoforms, and two JNK3 isoforms have been identified. JNK1 and JNK2 are expressed in most tissues, whereas JNK3 is expressed predominantly in neuronal cells [10–12]. JNK is involved in the regulation of various signaling pathways and plays an important role in cell proliferation, apoptosis, and differentiation [13]. Therefore, JNK has been implicated in a variety of human diseases, including tumors. Increasing evidence from animal and clinical studies suggests a critical role for aberrant activation of JNK in tumor development, and JNK is drawing increasing attention as a promising target of antitumor therapy in the near future [14, 15]. Recent studies have shown that the JNK signaling pathway plays an important role in the malignant progression of GBMs [16].

Ca^2+^ release-activated Ca^2+^ (CRAC) channels mediate Ca^2+^ influx in many cell types. These channels are formed by the tetraspanning plasma membrane proteins, named Orai1, Orai2, and Orai3. These ORAI proteins mediate Ca^2+^ influx by store-operated Ca^2+^ entry (SOCE) [17]. CRAC channels are hexameric complexes composed of individual or potentially multiple Orai homologues. The transmembrane domains are highly conserved between all three Orai homologues [18], and all the Orai homologues can function as Ca^2+^ channels when they are overexpressed. The properties of ectopically expressed Orai1, Orai2, and Orai3 channels are similar to those of endogenous CRAC channels, including activation by Ca^2+^ store depletion, high Ca^2+^ selectivity, inward rectification, and Ca^2+^-dependent inactivation (CDI). However, the three ORAI homologues differ in some of their channel properties, including fast and slow CDI and their sensitivity to pharmacological inhibitors such as 2-aminoethoxydiphenyl borate [19].

Recent results of our research team showed that Orai1 is upregulated in glioma specimens and glioma cell lines compared with nontumor control brain tissue, and the regulatory degree is positively correlated with the grades of gliomas. Further analysis found that the use of the inhibitor SKF96365 or RNAi downregulation of Orai1 significantly inhibited the invasion and migration of glioma cells and the epithelial-mesenchymal transition- (EMT-) like process. In vivo experiments showed that Orai1 can inhibit C6 colloidal downregulation of invasion and migration of tumor cells in a rat brain [20]. Motiani et al. found significant upregulation of Orai1 in primary GBM cell lines, and Orai1 knockdown significantly inhibited GBM cell proliferation and invasiveness [21]. However, as a homolog of Orai1, the role of Orai2 in gliomas has not yet been elucidated. In the present study, we aimed to evaluate the Orai2-related key genes and pathways by analyzing RNA-seq data across microarray chips including a total of 1231 glioma samples, and to the best of our knowledge, this is the largest and most comprehensive study of the main role and internal mechanism of Orai2 in GBM.

## 2. Methods

### 2.1. Samples and Patients

Transcriptome and clinical data of glioma patients were retrieved from The Cancer Genome Atlas (TCGA) dataset (http://cancergenome.nih.gov/); French and Sun datasets were available on R2 Genomics Analysis and Visualization Platform (http://r2.amc.nl or http://r2platform.com) and Microarray data of GEO (http://www.ncbi.nlm.nih.gov/geo); GSE23806 was enrolled in this study. GSE23806 profile included microarray data from 17 cases of neurospheres, 11 primary tumors (corresponding tumor tissue of glioblastoma patients), 27 cases of glioblastoma stem-like cell lines, and 36 cases of conventional glioma cell lines. The mRNA expression level of 539 GBM samples was measured through AffyU133a microarray platform. Meanwhile, 530 lower-grade glioma (WHO II and WHO III) and 172 GBM patients were measured through an Illumina HiSeq microarray platform. All of these data are available from the TCGA database. Both the French (284 cases included) and Sun (180 cases included) were acquired from R2 Genomics Analysis and Visualization Platform. Cluster analyses were performed and visualized using Cluster/Java Treeview. Patients were divided into the Orai2^low^ and Orai2^high^ groups by the mean expression levels. ChIPBase v2.0 (http://rna.sysu.edu.cn/chipbase/) was used to perform Pearson's correlation tests. The Retrieval of Interacting Genes/Proteins (STRING database v10.5) resource was utilized for PPI network analysis and prediction of protein-protein interactions.

### 2.2. Identification of DEGs

Morpheus, an R-associated web application, was applied to filtrate DEGs among four subtypes in GBM. The *P* ≤ 0.01 and ∣log FC∣⩾1 were considered the cutoff criterion. All results of DEGs were downloaded in text format, with hierarchical clustering analysis being conducted later in Morpheus (https://software.broadinstitute.org/morpheus/).

### 2.3. GO and Pathway Enrichment Analysis of DEGs

The online tool, Database for Annotation, Visualization and Integrated Discovery (DAVID, https://david.ncifcrf.gov/) provided comprehensive information for the list of genes by GO and KEGG pathway analyses. In addition, GO enrichment analysis included three different aspects: biological process (BP), molecular function (MF), and cellular component (CC) [22]. KEGG enrichment analysis was associated with genomic information's functional interpretation and practical application [23]. *P* < 0.05 was considered to indicate a statistically significant difference.

### 2.4. Statistical Analysis

In this study, the prognostic value of Orai2 was estimated by Kaplan-Meier analysis and Cox proportional hazard model analysis using SPSS statistical software (version 19). Other statistical computations and drawing of figures were performed with several packages (ggplot2, pheatmap, and circlize) in the statistical software environment R, version 3.3.2 (http://www.r-project.org). For all statistical methods, *P* < 0.05 was considered a significant difference.

## 3. Results

### 3.1. Survival Analysis of Orai2 Expression Status

**Figure 1:**
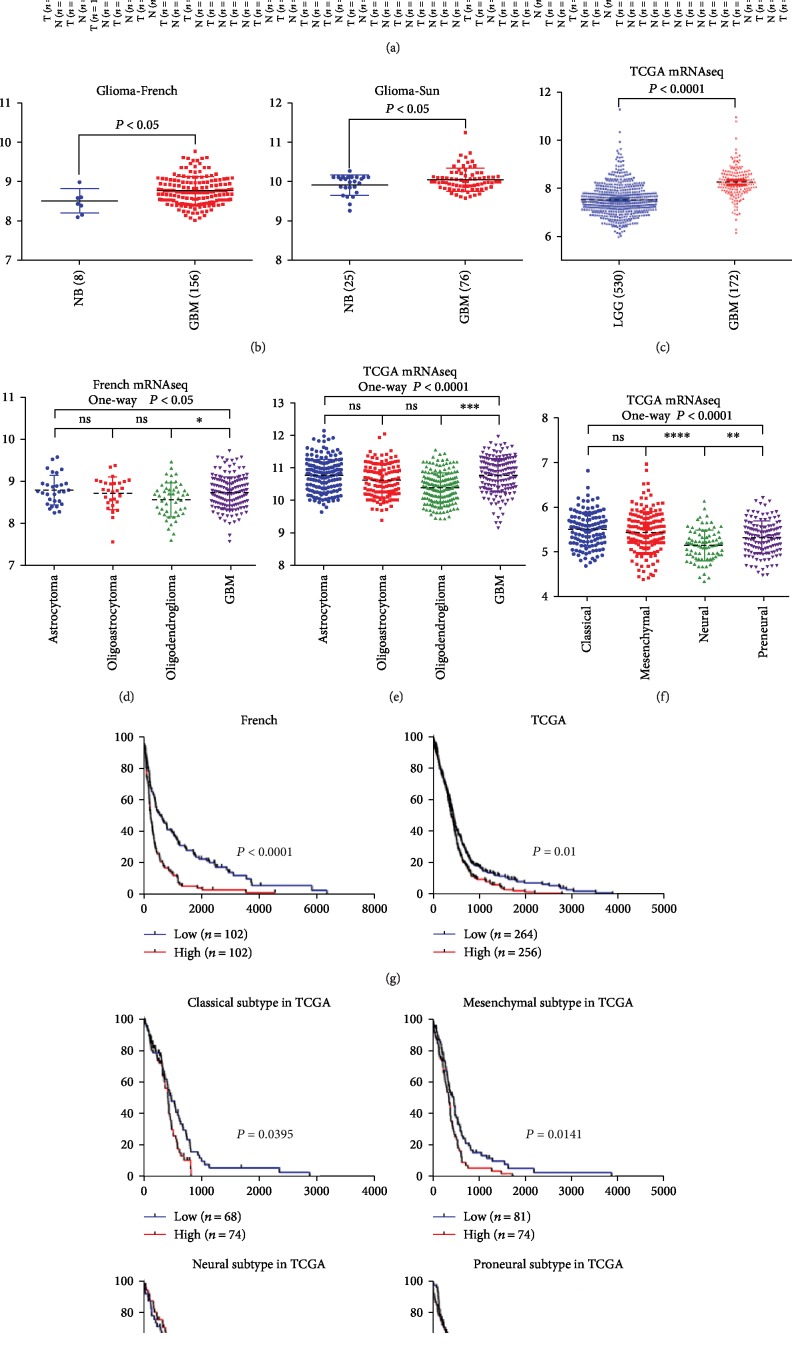
Orai2 expression was increased in GBM, and elevated Orai2 expression was a prognostic indicator of poor survival in glioma patients. (a) ORAI2 expression in various tumors from GEPIA (Gene Expression Profiling Interactive Analysis). (b, c) Orai2 level was increased in GBM compared with normal brain samples from the French, Sun, and TCGA datasets. NB: normal brain samples. (d, e) Compared with lower-grade glioma (WHO II and WHO III) and other glioma tissue types, Orai2 expression levels were higher in GBM (WHO IV). (f) The levels of Orai2 were analyzed in GBM tissues from the TCGA dataset; Orai2 was significantly upregulated in the classical and mesenchymal subtypes of GBM. (g, h) Kaplan-Meier survival curve analysis indicated that patients with higher Orai2 expression in the classical and mesenchymal subtypes had a shorter overall survival, while the other two subtypes had no prognostic value.

TCGA datasets retrieved from the GEPIA (Gene Expression Profiling Interactive Analysis) showed that among the several types of tumors that are common in humans, the tumors with the highest expression of Orai2 are glioblastoma multiforme (GBM), diffuse large B-cell lymphoma (DLBCL), acute myeloid leukemia (AML), pheochromocytoma and paraganglioma (PCPG), and thyroid carcinoma (THCA) (Figure 1(a)). We analyzed Orai2 mRNA expression in the French and Sun Brain datasets from the R2 analysis and visualization platform. The mRNA expression results showed that Orai2 was overexpressed in GBM samples compared with normal brain tissues (Student's *t*-test, *P* < 0.05) (Figure 1(b)). Compared with lower-grade glioma (WHO II and WHO III), Orai2 expression was significantly increased in GBM from the TCGA dataset (Student's *t*-test, *P* < 0.001) (Figure 1(c)). When we classified the data according to glioma tissue type in the French and TCGA datasets, we found that the expression level of Orai2 in GBMs was also higher than that of astrocytomas, oligoastrocytomas, and oligodendrogliomas. There was no significant difference in the expression of Orai2 in astrocytomas, oligoastrocytomas, and oligodendrogliomas (Figures 1(d) and 1(e)). To further confirm the Orai2 expression results in GBM, we analyzed data in TCGA. The results revealed that compared with other subtypes, Orai2 was significantly upregulated in the classical and mesenchymal subtypes of GBMs (Figure 1(f)). Accordingly, the Kaplan-Meier survival analysis revealed that patients with high levels of Orai2 had shorter overall survival (OS) times than patients with low levels of Orai2 in the French and TCGA datasets (Figure 1(g)). We evaluated the prognostic value of Orai2 by Kaplan-Meier survival curve analysis in four subtypes of GBM. The results showed that patients with higher Orai2 expression in the classical and mesenchymal subtypes had a shorter overall survival, while the other two subtypes had no prognostic value (Figure 1(h)).

### 3.2. Orai2 Expression Characteristics in Classical and Mesenchymal Subtypes of GBMs

**Figure 2:**
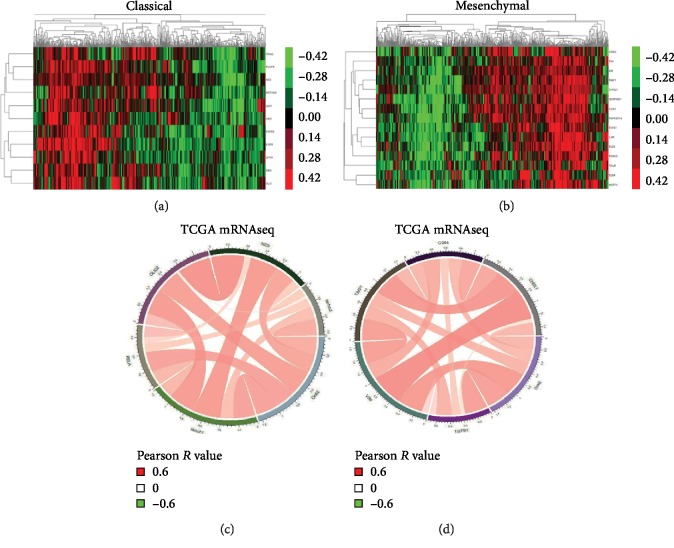
Orai2 expression characteristics in classical and mesenchymal subtypes of GBM cluster analysis and Pearson's correlation analysis in the Orai2 and (a, c) classical (b, d) and mesenchymal markers in the TCGA dataset.

By the analysis of the TCGA database, Verhaak et al. found that the classical type has astrocyte characteristics and expresses neural precursors and stem cell markers (NES, Notch pathway, and Sonic hedgehog pathway), and its tumor cells are highly proliferative; the mesenchymal type has astrocyte features, which mainly express mesenchymal markers (CHI3L1, CD44, and vimentin) and astrocyte markers (GFAP, Sox9, and CD44), the high activity of their markers often suggests epithelial-mesenchymal transition (EMT), and necrotic and inflammatory reactions often occur in tumors; classical and mesenchymal subtypes of GBM patients have a poor prognosis [9, 24]. Orai2 RNA levels were strongly positively associated with classical subgroup markers (NOTCH1, NES, NOTCH1, RELA, and OLIG2) (Figures 2(a) and 2(c)) and mesenchymal subgroup markers (TGFB1, VIM, TIMP1, CD44, and CHI3L1) (Figures 2(b) and 2(d)). These results indicate that Orai2 is strongly associated with the classical and mesenchymal phenotypes in human GBMs.

### 3.3. Orai2 Clinical Expression Status

In the TCGA dataset (*n* = 539), high expression of Orai2 was selectively associated with elder ages at first-time diagnosis and shorter overall survival years, days to progression, and days to recurrence. Furthermore, higher Orai2 expression was obviously related to shorter overall survival years and temozolomide (TMZ) chemoradiation and long-term therapy with TMZ in classical patients. Meanwhile, higher Orai2 expression was distinctly associated with shorter overall survival years and GBM recurrence in the mesenchymal samples (Supplementary [Supplementary-material supplementary-material-1]). The above results suggest that Orai2 may be involved in the recurrence of GBMs and could be a new diagnostic and clinical prognostic biomarker, especially in the classical and mesenchymal types of GBMs.

### 3.4. Identification and Hierarchical Clustering Analysis of DEGs

**Figure 3:**
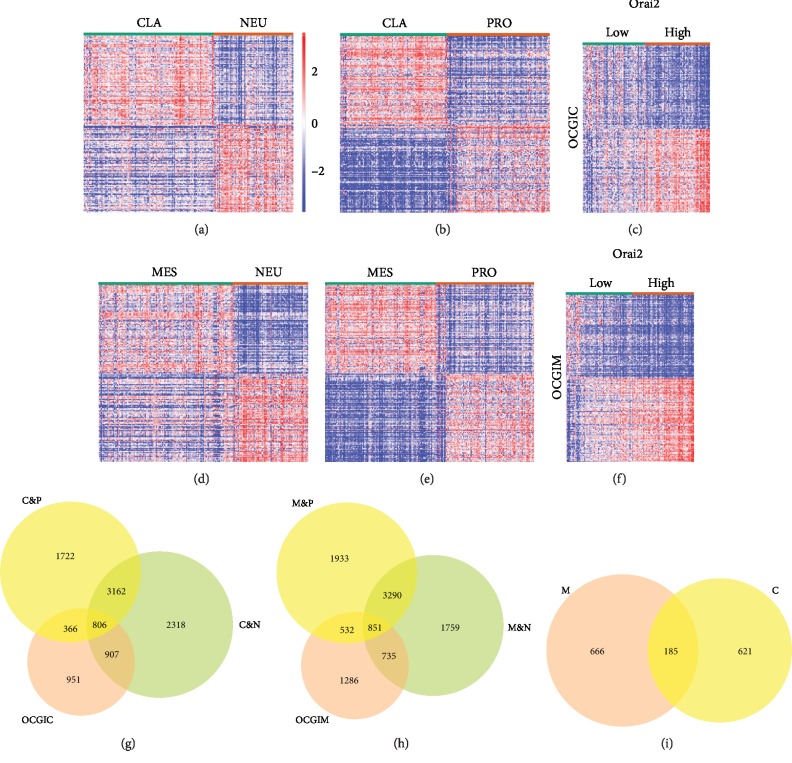
Orai2 differential expression analysis. The heat maps displaying the DEGs in the CLA, MES, (a, d) NEU, and (b, e) PRO subtypes of GBM. (c, f) Heat map displaying the DEGs between the Orai2^high^ and Orai2^low^ groups in CLA and MES GBM. (g) A comparison of the 3404, 7193, and 6056 genes revealed 806 common genes specific to the Orai2 that were correlated with the classical subgroup. (h) A comparison of the 3030, 6635, and 6606 genes revealed 851 common genes specific to the Orai2 that were correlated with the mesenchymal subgroup. (i) A comparison of the 806 and 851 genes revealed 185 common genes specific to Orai2 that were correlated between the classical and mesenchymal subgroups. C&N: the overlap between the classical and neural subtypes; C&P: the overlap between the classical and proneural subtypes; M&N: the overlap between the mesenchymal and neural subtypes; M&P: the overlap between the mesenchymal and proneural subtypes; OCGIC: Orai2-correlated genes in the classical GBM; OCGIM: Orai2-correlated genes in the mesenchymal GBM; GBM: glioblastoma; DEG: differentially expressed genes.

First, a total of 7193, 6056, 6635, and 6606 DEGs were obtained separately from TCGA datasets between classical or mesenchymal and other subtypes of GBM with the criteria of *P* < 0.05 and ∣log FC∣⩾1.0. Hierarchical clustering analysis was conducted through Morpheus, a web-based online tool, with the series matrix data of the DEGs. The heat map is shown in Figures 3(a)–3(e) (the top 100 genes). Next, 3404 and 3030 genes were acquired from classical and mesenchymal GBMs by the mean Orai2 expression (Figures 3(c) and 3(f)). Finally, we summarized the abovementioned hub genes that overlapped among groups to identify DEGs, yielding a total of 185 genes (Figures 3(g)–3(i)).

### 3.5. Orai2-Related Biological Processes

**Figure 4:**
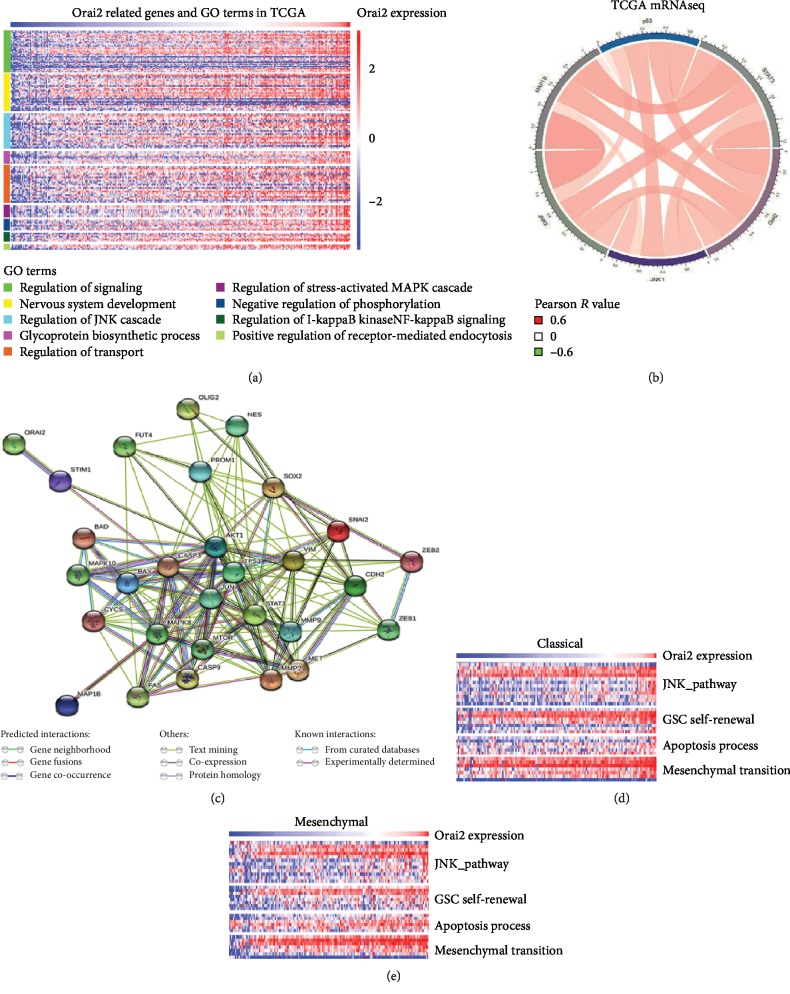
Orai2-related biological process. Orai2-related biological process by (a) gene ontology analysis in TCGA datasets. (b) Association between Orai2 and the main proteins of the JNK pathway in TCGA. Red ribbons indicate positive correlation of two terms while green ribbons indicate negative correlation. The width of ribbon and scale of colors indicate correlation coefficient. Association in Orai2 expression and GSC self-renewal, apoptosis, and EMT-related molecules in (d) classical and (e) mesenchymal TCGA datasets. (c) The PPI network also confirmed these findings.

For the purpose of deeply understanding the function of the identified DEGs, GO and KEGG analyses were performed separately in DAVID. The results are displayed in Supplementary [Supplementary-material supplementary-material-1]. We found that Orai2 was mainly involved in the regulation of signaling, nervous system development, regulation of the JNK cascade, glycoprotein biosynthetic process, regulation of transport, regulation of the stress-activated MAPK cascade, negative regulation of phosphorylation, regulation of I-kappaB kinase/NF-kappaB signaling, and positive regulation of receptor-mediated endocytosis (Figure 4(a)). To investigate whether Orai2 expression is correlated with JNK pathway-related molecule expression, we analyzed data in TCGA and found that Orai2 was correlated with JNK pathway-related molecules in the classical and mesenchymal subtypes of GBM samples (Figure 4(b)). To further investigate the downstream mechanism of Orai2 in regulating the JNK pathway, we mapped the interactome of Orai2 with GSC self-renewal, apoptosis process, and EMT-associated proteins by using STRING (https://string-db.org) (Figure 4(c)). Furthermore, we evaluated the association between the JNK pathway and GSC self-renewal, apoptosis, and EMT from TCGA transcriptomic data. A strong correlation between Orai2 and GSC self-renewal, apoptosis, and EMT was observed (Figures 4(d) and 4(e)).

### 3.6. Association of Orai2 and Self-Renewal, Apoptosis, and EMT

**Figure 5:**
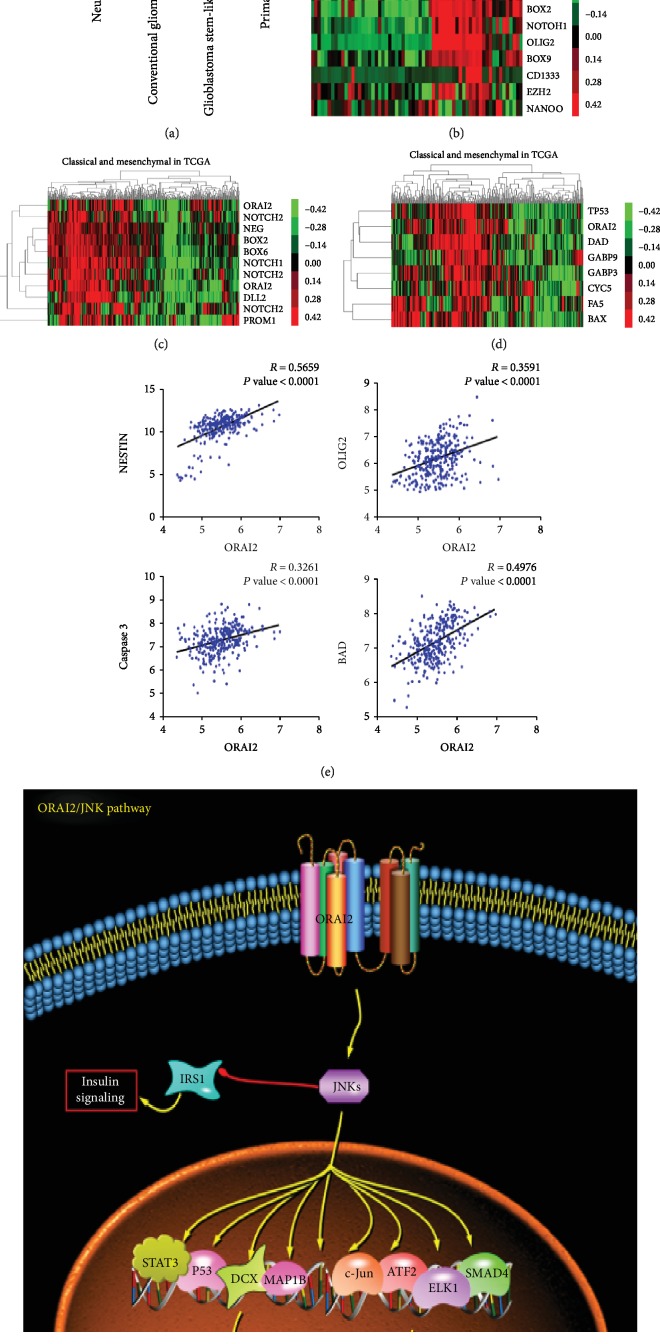
Orai2 was associated with stemness and apoptosis via the JNK pathway. (a) Orai2 expression is relatively high in glioblastoma stem-like cell lines and neurospheres compared to conventional glioma cell lines and primary tumors from GEO profiles (GEO: GDS3885). (b) Expression heat map of Orai2 in GSC lines (*n* = 27) and NSTC lines (*n* = 36) from GEO profiles (GEO: GDS3885). Orai2 was significantly upregulated in GSCs relative to the nontumor stem cells (NSTC). Cluster analysis and Pearson's correlation analysis in the Orai2 and (c, e) stemness and (d, e) apoptosis markers in TCGA datasets. (f) Schematic mechanism of the Orai2/JNK pathway signaling axis.

One GEO database (GSE23806) was used to analyze the expression of Orai2 in glioblastoma stem-like cell lines, conventional glioma cell lines, neurospheres, and primary tumors (corresponding tumor tissue of glioblastoma patients). We found that Orai2 expression was relatively high in glioblastoma stem-like cell lines and neurospheres compared to conventional glioma cell lines and primary tumors (Figure 5(a)). At the same time, we utilized another independent GEO microarray dataset (GDS3885) and found that Orai2 was closely related to stem-related genes that were significantly upregulated in GSCs relative to the matched nontumor stem cells (NSTCs) (Figure 5(b)). In addition, to test Orai2 as a biomarker and a regulator of glioma stem cells, we further found a relationship between Orai2 and GSC markers, such as PROM1, NES, SOX2, OLIG2, NOTCH1, SOX9, MET, NOTCH2, and DLL3. Orai2 was obviously associated with GSC markers (NESTIN, *R* = 0.0.5659; OLIG2, *R* = 0.3591) (Figures 5(c) and 5(e)). These results suggested that Orai2 may serve as a biomarker or a regulator of glioma stem cells. Similarly, we clustered the mRNA microarray data from TCGA and found that Orai2 is significantly related to apoptosis-related proteins in the classical and mesenchymal types of GBM. Likewise, we analyzed the association between Orai2 and apoptosis markers, such as TP53, CASP3, CASP9, CYCS, BAX, FAS, and BAD. Orai2 was positively correlated with apoptosis markers (caspase 3, *R* = 0.3261; BAD, *R* = 0.4976) (Figures 5(d) and 5(e)). These results suggested that Orai2 may integrate apoptosis in the classical and mesenchymal types of GBM. To explore whether Orai2 is involved in EMT-like in classical and mesenchymal subtypes of GBM, we calculated the relationship between Orai2 and EMT-related markers by cluster analysis (Supplementary Fig. [Supplementary-material supplementary-material-1]) and Pearson's correlation analysis (Supplementary Fig. [Supplementary-material supplementary-material-1]). The results showed that orai2 was negatively correlated with epithelial marker CDH1 (E-cadherin) expression (CDH1^CLA^, *R* = ‐0.0203; CDH1^MES^, *R* = ‐0.1934) (Supplementary Fig. [Supplementary-material supplementary-material-1]), but positively correlated with mesenchymal markers such as NCOR1/VIM/ZEB1/PTN/CDH2 (N-cadherin). (NCOR1^CLA^, *R* = 0.4860; VIM^CLA^, *R* = 0.4264; ZEB1^CLA^, *R* = 0.3265; PTN^CLA^, *R* = 0.3942; CDH2^CLA^, *R* = 5662; NCOR1^MES^, *R* = 0.4970; VIM^MES^, *R* = 0.5689; ZEB1^MES^, *R* = 0.4572; PTN^MES^, *R* = 0.4239; CDH2^MES^, *R* = 0.5821) (Supplementary Fig. [Supplementary-material supplementary-material-1]). These results suggested that Orai2 is likely to be involved in the EMT-like process in GBM and plays a facilitating role. Finally, schematic of the Orai2/JNK signaling pathway is shown in Figure 5(f).

## 4. Discussion

In summary, this is the largest and most comprehensive study identifying the key pathways and genes in Orai2-mediated GBM by bioinformatic analyses. We revealed that Orai2 expression was significantly higher in GBM (WHO IV) than lower-grade glioma (WHO II and WHO III). Patients with high expression of Orai2 have a poor prognosis compared with low expression of Orai2 in GBM patients. Furthermore, we found that the expression level of orai2 mRNA in the classical and mesenchymal types of GBM was significantly higher than that of other subtypes. Genes associated with Orai2 were mainly involved in GSC self-renewal, apoptosis, and EMT. In clinical terms, higher Orai2 expression was independently associated with a worse prognosis of patients with GBM, especially in the classical and mesenchymal types of GBM.

Our group has previously studied and found the expression characteristics of orai1 in gliomas across all grades in 61 patients [20]. In this study, we collected and characterized orai2 expression levels in 1231 glioma samples from four large independent databases. We found that the mRNA levels of Orai2 were higher in GBM than in nonneoplastic brain tissues, indicating that Orai2 acted as a tumor promoter in GBM. Notably, our findings showed that Orai2 was expressed at relatively higher levels in the classical and mesenchymal subtypes. At the same time, we also found that Orai2 RNA levels were strongly positively associated with classical and mesenchymal subgroup markers. We concluded that Orai2 is strongly associated with the classical and mesenchymal phenotypes in human GBMs.

In this study, GO term enrichment and KEGG pathway analysis results revealed that Orai2 was involved in and regulated GSC self-renewal, apoptosis, and EMT. Simultaneously, according to our results, Orai2 was likely to regulate these biological processes through the JNK pathway. This hypothesis requires in vitro and in vivo experiments to further confirm it in the near future.

Some studies have shown that JNK, as a key molecule in the maintenance of GSC, may be a feasible molecular target for glioma [14, 15]. Moreover, related studies have suggested that JNK plays a pivotal role in other malignant tumors, such as ovarian, pancreatic, and lung cancers. Okada et al. found that JNK inhibitors could prevent pancreatic tumor formation via eliminating cancer stem-like cells [25–27]. JNK has been extensively studied in the induction of apoptosis. Upon activation, phosphorylated JNK translocates to the nucleus where it phosphorylates and regulates transcription factors such as c-Jun, p53, and c-Myc, which are involved in the induction of cell apoptosis [28]. The alkylating anticancer drugs temozolomide (TMZ) and nimustine (ACNU) induce apoptosis in glioma cells at late times following treatment by activating JNK and c-Jun [29].

Interestingly, other studies have confirmed that the JNK inhibitor SP600125 was sufficient to inhibit cell viability, migration, and invasion of GBM cells through decreasing the phosphorylation levels of both c-Jun and Akt [30]. Combined inhibition of PI3K and JNK can inhibit the invasion and migration of glioblastoma and lamellipodia and membrane ruffle formation [31]. Likewise, Ueno et al. found that knocking down JNK in TMZ-resistant GBM cells could enhance invadopodia formation through the JNK-paxillin axis to accelerate ECM degradation [32].

However, to date, there have been few reports on the specific mechanism of action of Orai2 in glioblastoma, so our bioinformatics analysis of Orai2 is very meaningful. We have shown through bioinformatics analysis that orai2 may participate in the JNK pathway to exert antitumor effects, which provides direction for future research, and we will further test the relationship between these molecules. We hoped that these and subsequent related experiments could benefit GBM patients through targeting Orai2/JNK-related factors in the near future.

## Data Availability

The data used to support the findings of this study are included within the article.

## Supplementary Materials

Supplementary Materials Supplementary Fig. 1: cluster analysis and Pearson's correlation analysis between the Orai2 and EMT markers in (A, C) classical and (B, D) mesenchymal TCGA datasets. Supplementary Table I: clinical and molecular pathology features of TCGA GBM samples in association with Orai2 expression. Supplementary Table II: gene ontology and KEGG pathway analysis of Orai2-correlated genes in classical and mesenchymal GBM.

## References

[1] Kang S., Hong J., Lee J. M., Moon H. E., Jeon B., Choi J. (2017). Trifluoperazine, a well-known antipsychotic, inhibits glioblastoma invasion by binding to calmodulin and disinhibiting calcium release channel IP_3_R. *Molecular Cancer Therapeutics*.

[2] Stupp R., Taillibert S., Kanner A. A. (2015). Maintenance therapy with tumor-treating fields plus temozolomide vs temozolomide alone for glioblastoma: a randomized clinical trial. *JAMA*.

[3] Iser I. C., Pereira M. B., Lenz G., Wink M. R. (2017). The epithelial-to-mesenchymal transition-like process in glioblastoma: an updated systematic review and in silico investigation. *Medicinal Research Reviews*.

[4] Li Y., Wang W., Wang F. (2017). Paired related homeobox 1 transactivates dopamine D2 receptor to maintain propagation and tumorigenicity of glioma-initiating cells. *Journal of Molecular Cell Biology*.

[5] Segerman A., Niklasson M., Haglund C. (2016). Clonal variation in drug and radiation response among glioma-initiating cells is linked to proneural-mesenchymal transition. *Cell Reports*.

[6] Kalluri R., Weinberg R. A. (2009). The basics of epithelial-mesenchymal transition. *The Journal of Clinical Investigation*.

[7] Kast R. E., Skuli N., Karpel-Massler G., Frosina G., Ryken T., Halatsch M. E. (2017). Blocking epithelial-to-mesenchymal transition in glioblastoma with a sextet of repurposed drugs: the EIS regimen. *Oncotarget*.

[8] Louis D. N., Perry A., Reifenberger G. (2016). The 2016 World Health Organization classification of tumors of the central nervous system: a summary. *Acta Neuropathologica*.

[9] Verhaak R. G. W., Hoadley K. A., Purdom E. (2010). Integrated genomic analysis identifies clinically relevant subtypes of glioblastoma characterized by abnormalities in *PDGFRA*, *IDH1*, *EGFR*, and *NF1*. *Cancer Cell*.

[10] Sabio G., Davis R. J. (2014). TNF and MAP kinase signalling pathways. *Seminars in Immunology*.

[11] Kim E. K., Choi E. J. (2015). Compromised MAPK signaling in human diseases: an update. *Archives of Toxicology*.

[12] Kumar A., Singh U. K., Kini S. G. (2015). JNK pathway signaling: a novel and smarter therapeutic targets for various biological diseases. *Future Medicinal Chemistry*.

[13] Sehgal V., Ram P. T. (2013). Network motifs in JNK signaling. *Genes & Cancer*.

[14] Matsuda K. I., Sato A., Okada M. (2012). Targeting JNK for therapeutic depletion of stem-like glioblastoma cells. *Scientific Reports*.

[15] Kitanaka C., Sato A., Okada M. (2013). JNK signaling in the control of the tumor-initiating capacity associated with cancer stem cells. *Genes & Cancer*.

[16] Bubici C., Papa S. (2014). JNK signalling in cancer: in need of new, smarter therapeutic targets. *British Journal of Pharmacology*.

[17] Prakriya M., Lewis R. S. (2015). Store-operated calcium channels. *Physiological Reviews*.

[18] Hou X., Pedi L., Diver M. M., Long S. B. (2012). Crystal structure of the calcium release-activated calcium channel Orai. *Science*.

[19] Vaeth M., Yang J., Yamashita M. (2017). ORAI2 modulates store-operated calcium entry and T cell-mediated immunity. *Nature Communications*.

[20] Zhu M., Chen L., Zhao P. (2014). Store-operated Ca^2+^ entry regulates glioma cell migration and invasion via modulation of Pyk2 phosphorylation. *Journal of Experimental & Clinical Cancer Research*.

[21] Motiani R. K., Hyzinski-García M. C., Zhang X. (2013). STIM1 and Orai1 mediate CRAC channel activity and are essential for human glioblastoma invasion. *Pflügers Archiv - European Journal of Physiology*.

[22] Gaudet P., Škunca N., Hu J. C., Dessimoz C., Dessimoz C., Škunca N. (2017). Primer on the gene ontology. *The Gene Ontology Handbook*.

[23] Kanehisa M., Furumichi M., Tanabe M., Sato Y., Morishima K. (2017). KEGG: new perspectives on genomes, pathways, diseases and drugs. *Nucleic Acids Research*.

[24] Brennan C. W., Verhaak R. G. W., McKenna A. (2013). The somatic genomic landscape of glioblastoma. *Cell*.

[25] Okada M., Shibuya K., Sato A. (2013). Specific role of JNK in the maintenance of the tumor-initiating capacity of A549 human non-small cell lung cancer cells. *Oncology Reports*.

[26] Okada M., Shibuya K., Sato A. (2014). Targeting the K-Ras - JNK axis eliminates cancer stem-like cells and prevents pancreatic tumor formation. *Oncotarget*.

[27] Seino M., Okada M., Shibuya K. (2014). Requirement of JNK signaling for self-renewal and tumor-initiating capacity of ovarian cancer stem cells. *Anticancer Research*.

[28] Alapati K., Kesanakurti D., Rao J. S., Dasari V. R. (2014). uPAR and cathepsin B-mediated compartmentalization of JNK regulates the migration of glioma-initiating cells. *Stem Cell Research*.

[29] Tomicic M. T., Meise R., Aasland D. (2015). Apoptosis induced by temozolomide and nimustine in glioblastoma cells is supported by JNK/c-Jun-mediated induction of the BH3-only protein BIM. *Oncotarget*.

[30] Lopez-Bergami P., Kim H., Dewing A., Goydos J., Aaronson S., Ronai Z. (2010). c-Jun regulates phosphoinositide-dependent kinase 1 transcription: implication for Akt and protein kinase C activities and melanoma tumorigenesis. *The Journal of Biological Chemistry*.

[31] Zhao H. F., Wang J., Jiang H. R., Chen Z. P., To S. S. T. (2016). PI3K p110*β* isoform synergizes with JNK in the regulation of glioblastoma cell proliferation and migration through Akt and FAK inhibition. *Journal of Experimental & Clinical Cancer Research*.

[32] Ueno H., Tomiyama A., Yamaguchi H. (2015). Augmentation of invadopodia formation in temozolomide-resistant or adopted glioma is regulated by c-Jun terminal kinase-paxillin axis. *Biochemical and Biophysical Research Communications*.

