# Supplementary material for: Identification of Key Pathways and Genes in the Orai2 Mediated Classical and Mesenchymal Subtype of Glioblastoma by Bioinformatic Analyses
Source: Dis Markers. 2019 Oct 20;2019:7049294. doi: 10.1155/2019/7049294 (PMC6855003; doi:10.1155/2019/7049294)
Supplement: Supplementary Materials — Supplementary Fig. 1: cluster analysis and Pearson's correlation analysis between the Orai2 and EMT markers in (A, C) classical and (B, D) mesenchymal TCGA datasets. Supplementary Table I: clinical and molecular pathology features of TCGA GBM samples in association with Orai2 expression. Supplementary Table II: gene ontology and KEGG pathway analysis of Orai2-correlated genes in classical and mesenchymal GBM. [file 7049294.f1.docx]

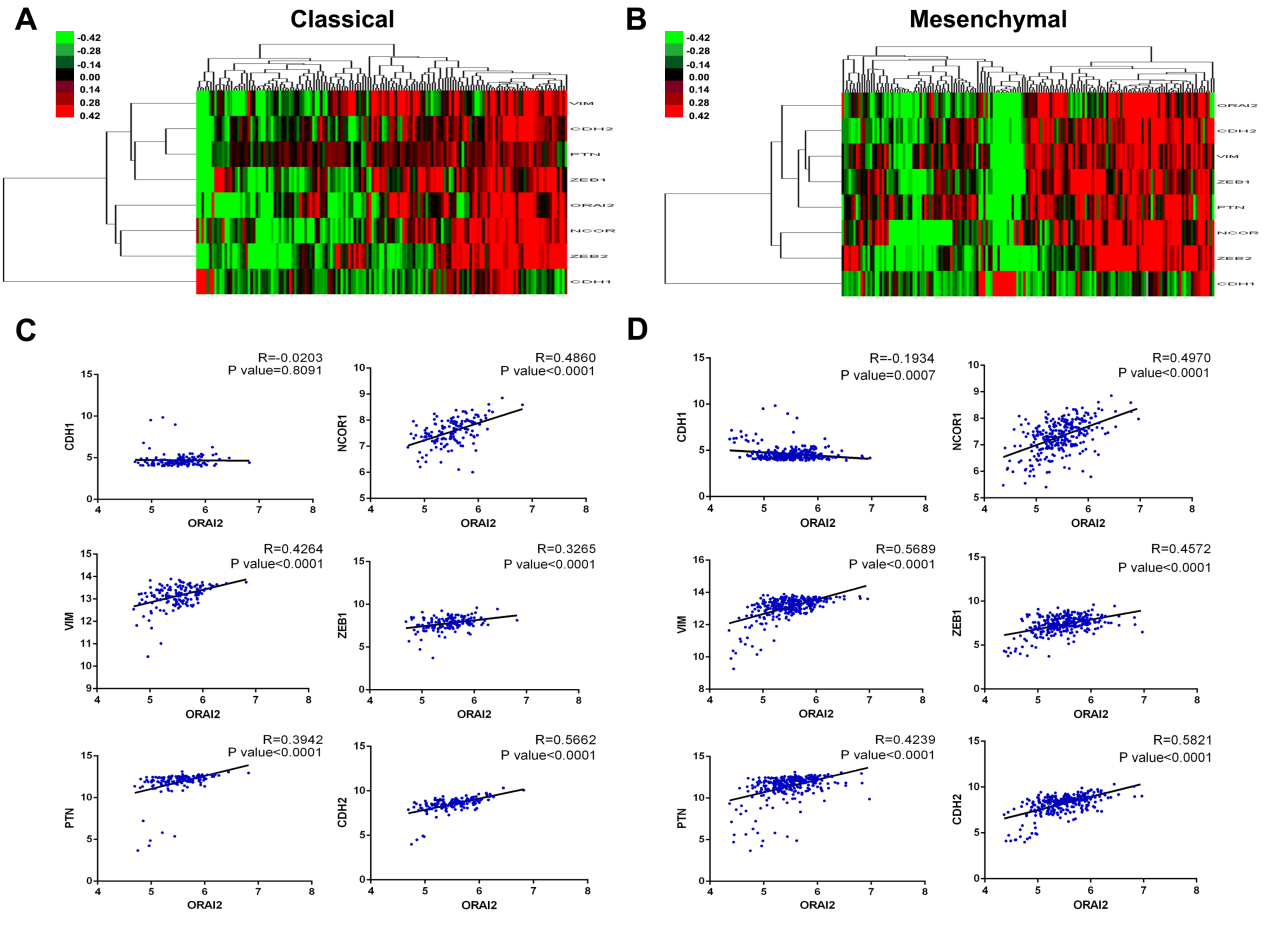


**Supplementary Fig. 1** Cluster analysis and Pearson correlation analysis between the Orai2 and EMT markers in (A,C) classical and (B,D) mesenchymal TCGA datasets.

| Variables | Low | | | | | High | | | | | P‑value | | | | |
| --- | --- | --- | --- | --- | --- | --- | --- | --- | --- | --- | --- | --- | --- | --- | --- |
| Subtype | Total | CLA | MES | NEU | PRO | Total | CLA | MES | NEU | PRO | Total | CLA | MES | NEU | PRO |
| Age (years ± SD) | 55±15 | 56±14 | 58±11 | 59±13 | 51±17 | 60±13 | 60±12 | 61±12 | 60±13 | 56±17 | 0.000^a^ | 0.063^a^ | 0.093^a^ | 0.789^a^ | 0.079^a^ |
| Gender, female/male | 108/158 | 32/38 | 27/54 | 16/27 | 28/38 | 97/162 | 29/45 | 31/44 | 14/30 | 28/44 | 0.459^b^ | 0.428^b^ | 0.301^b^ | 0.596^b^ | 0.672^b^ |
| OS (days ± SD) | 543±599 | 514±488 | 501±556 | 520±535 | 651±754 | 382±358 | 357±240 | 331±297 | 386±282 | 459±561 | 0.000^a^ | 0.014^a^ | 0.019^a^ | 0.146^a^ | 0.090^a^ |
| KPS ≥70/<70 | 158/44 | 39/10 | 51/13 | 24/8 | 41/13 | 139/51 | 41/16 | 38/15 | 22/8 | 41/12 | 0.242^b^ | 0.360^b^ | 0.313^b^ | 0.880_b_ | 0.861^b^ |
| surgical resection,yes/no | 227/38 | 56/14 | 68/13 | 36/7 | 61/5 | 231/27 | 66/7 | 69/6 | 39/5 | 63/8 | 0.179^b^ | 0.078^b^ | 0.124^b^ | 0.506^b^ | 0.461^b^ |
| TMZChemoradiation.TMZChemoLong,yes/no | 94/126 | 21/39 | 28/38 | 16/17 | 28/29 | 102/99 | 33/29 | 29/28 | 17/17 | 24/28 | 0.099^b^ | 0.042^b^ | 0.348^b^ | 0.901^b^ | 0.756^b^ |
| Days to progression ± SD | 318±395 | 249±267 | 257±222 | 294±264 | 435±538 | 216±205 | 206±148 | 234±294 | 276±188 | 242±442 | 0.019^a^ | 0.435^a^ | 0.717^a^ | 0.815^a^ | 0.153^a^ |
| Days to recurrence ± SD | 488±566 | 359±353 | 299±218 | 455±633 | 718±782 | 265±254 | 346±223 | 116±85 | 308±81 | 407±546 | 0.012^a^ | 0.906^a^ | 0.008^a^ | 0.620^a^ | 0.234^a^ |
| G_CIMP_STATUS, yes/no | 32/229 | 1/68 | 2/78 | 1/41 | 24/40 | 14/246 | 0/75 | 0/75 | 0/44 | 18/54 | 0.0056^b^ | 0.9666^b^ | 0.5053^b^ | 0.4883^b^ | 0.1152^b^ |

**Supplementary Table I.** Clinical and molecular pathology features of TCGA GBM samples in association with Orai2 expression. aStudent's t‑test; bχ² test or Fisher's exact test; cLog‑rank test. KPS, Karnofsky Performance Scale; OS, overall survival; GBM, glioblastoma multiforme; TCGA, The Cancer Genome Atlas; SD, standard deviation. The level of Orai2 was classified by the mean value.

| Category | Term, function | Count | Percentage | P‑value | Genes |
| --- | --- | --- | --- | --- | --- |
| GOTERM_BP_FAT | GO:0023051~regulation of signaling | 39 | 1.41*10^-1^ | 2.51E-04 | CPLX3, SIPA1, NLRX1, MEN1, TNFRSF1A, ZGPAT, TMEM127, IFT122, SPRED2, PKD2, GDF9, TRAF6, EHD3, IQSEC1, CFLAR, DVL3, IRS2, PFKL, ACTN4, CD63, BICD1, STAT3, DNMBP, NOTCH2, DLX2, DUSP3, CNIH3, RIPK1, CA7, DLX5, SIPA1L1, NEUROD2, SMPD1, TNNI3K, MCC, TRIP6, FAIM2, MAP3K11, RNF41 |
| GOTERM_BP_FAT | GO:0007399~nervous system development | 26 | 9.44*10^-2^ | 7.51*10^-3^ | SSH1, ARNT2, KCNIP2, MTMR2, IFT122, PKD2, TRAF6, KIF13B, FZD9, DVL3, IRS2, GLRB, KIF3A, SMAD1, KIDINS220, STAT3, WEE1, DLX2, SIPA1L1, DLX5, NEUROD2, MNX1, CYFIP1, CTNS, POFUT1, FAIM2 |
| GOTERM_BP_FAT | GO:0046328~regulation of JNK cascade | 6 | 2.17*10^-2^ | 1.05*10^-2^ | MEN1, DVL3, DUSP3, RIPK1, TRAF6, MAP3K11 |
| GOTERM_BP_FAT | GO:0032872~regulation of stress-activated MAPK cascade | 6 | 2.17*10^-2^ | 2.13*10-2 | MEN1, DVL3, DUSP3, RIPK1, TRAF6, MAP3K11 |
| GOTERM_BP_FAT | GO:0043122~regulation of I-kappaB kinase/NF-kappaB signaling | 6 | 2.17*10^-2^ | 4.27*10^-2^ | CFLAR, TNFRSF1A, RIPK1, NLRX1, TRIP6, TRAF6 |
| GOTERM_CC_FAT | GO:0048471~perinuclear region of cytoplasm | 11 | 3.99*10^-2^ | 2.85*10^-3^ | FZD9, GALNT2, OSBPL3, ACTN4, CYFIP1, HECTD3, TLK2, TRAF6, EHD1, EHD3, BICD1 |
| GOTERM_CC_FAT | GO:0005924~cell-substrate adherens junction | 10 | 3.63*10^-2^ | 4.10*10^-3^ | TRIOBP,LRP1, CAPN5, ACTN4, GIT2, CYFIP1, ZYX, TRIP6, EHD3, PXN |
| GOTERM_MF_FAT | GO:0008376~acetylgalactosaminyltransferase activity | 4 | 1.45*10^-2^ | 1.09*10^-3^ | GALNT2, GALNT10, CHPF, GALNT11 |
| GOTERM_MF_FAT | GO:0004725~protein tyrosine phosphatase activity | 5 | 1.81*10^-2^ | 1.22*10^-2^ | MTMR2, DUSP3, PTPN9, SSH1, PTPN12 |
| KEGG_PATHWAY | cfa04142:Lysosome | 9 | 3.26*10^-2^ | 8.29E-05 | SGSH, GNS, NAGPA, GALNS, SMPD1, ACP2, CD63, CTNS, AP3B1 |
| KEGG_PATHWAY | cfa00512:Mucin type O-Glycan biosynthesis | 4 | 1.45*10^-2^ | 5.65*10^-3^ | GALNT2, GALNT10, GALNT11, B4GALT5 |
| KEGG_PATHWAY | cfa00531:Glycosaminoglycan degradation | 3 | 1.08*10^-2^ | 2.43*10^-2^ | SGSH, GNS, GALNS |
| KEGG_PATHWAY | cfa04144:Endocytosis | 8 | 2.90*10^-2^ | 3.27*10^-2^ | GIT2,EEA1,TRAF6, EHD1, IQSEC1, EHD3, EPN2, RNF41 |

**Supplementary Table II.** Gene ontology and KEGG pathway analysis of Orai2 correlated genes in classical and mesenchymal GBM.
